# Practical Notes and Formulæ

**Published:** 1888-12

**Authors:** 


### PRACTICAL NOTES AND FORMULÆ.

**Diarrhœa.**—In a case of obstinate diarrhœa in a child Dr. Reynolds (*Medical and Surgical Reporter*) after failure of a number of remedies says: I now ordered cloths wrung out of hot water to be applied to the abdomen, and the following prescription to be used:

| | | |
|---|---|---|
| ℞. | Plumbi acetat | gr. iss, |
| | Tr. opii | gtt. x, |
| | Spr. vini Gallici | f ʒ ii, |
| | Aquæ | q. s. ad f ℥ j. |

M. Sig.—A teaspoonful once in three hours.

I also ordered small powders of pepsin and ingluvin to be given after each time the baby took its nourishment. August 2, I found the temperature 101°, the bowels free from gas, and that only four movements of the bowels had taken place since I last saw it, while all the symptoms showed improvement. The last prescription was now given only one in four hours, and a few drops of brandy were given between the doses, so that the infant got a little stimulant once in two hours. I also gave ½ grain of quiniæ sulph. once in four hours. August 3, I found the child's temperature normal; that it had only two discharges from the bowels in twenty-four hours; that its mouth and tongue were becoming moist, and that it was showing an inclination for food. I therefore discontinued the quinine and pepsin. On the next day finding my patient still improving, I prescribed a tonic of cinchona and iron, to be continued for several days, and the child has now made a perfect recovery.

**Treatment of Comedones.**—After I had administered ether to a patient who was greatly affected with comedones, I noticed that they were easily pressed out, due, perhaps, to the solvent properties of ether on these greasy concretions. I resolved to give the ether treatment a fair trial. I used the following on several cases with gratifying results:

| | | |
|---|---|---|
| ℞. | Sulphuric ether | ℥ j, |
| | Carbonate of ammonia | ʒ j, |
| | Boracic acid | gr. xx, |
| | Water, to make | ℥ ij. |

M.

Apply twice a day. The carbonate of ammonia, with the grease forms a soap. The boracic acid acts as an antiseptic, and the ether as a solvent.—*Medical Analectic.*

**Substitute for Cod-Liver Oil.**—The following combination is highly recommended by Lamande for delicate stomachs which refuse cod-liver oil:

℞. Glycerini .................................. ℥ x,

Tinct. iodi .................................. ʒ ss,

Potass. iodidi .................................. grs. xij.

M. Sig.—A teaspoonful fifteen minutes before meals.—*Gazette Medicale.*

**Pomade Against Neuralgia**.—(*Medical Age.*)

℞. Menthol .................................. gr. xv,
Cocaine .................................. gr. v,
Hydrate of chloral .................................. gr. iij,
Vaseline .................................. ʒ j.

M.

**Tetanus.**—

℞. Chloral. hydrat .................................. ʒ iij,

Potass. bromid .................................. ʒ iij,

Aquæ font .................................. ℥ ij.

M. Sjg.—A teaspoonful every half-hour.—*Dr. Eliot in Medical and Surgical Reporter.*

**Epilepsy.**—(*Medical and Surgical Reporter.*)

℞. Potass. bromid .................................. ℥ i,
Sodii brom .................................. ℥ i,
Tinct. belladon .................................. f ℥ ss,
Acquæ .................................. q. s. ad f ℥ vj.

M. Sig.—Teaspoonful after each meal. To be increased.

**Whooping Cough.**—(*Medical World.*)

℞. Atropiæ sulphat .................................. gr xvj,

Syr. ipecacuanhæ .................................. ʒ j,

Sy. totulani .................................. ʒ j,

Aq. dest. ad .................................. ℥ ij.

One teaspoonful every one to three hours.

℞. Potass. bromid .................................. gr. iv,

Ext. belladon .................................. gr. ¼,

Syr. papaveris .................................. *m* xv,

Aq. dest. ad .................................. ʒ ij.

Every four hours. For children over four in whooping cough.

**Pleurisy.**—

℞. Potassii iodidi .................................. gr. lxxx,

Ammoniæ carb .................................. ʒ j,

Tinct. cinchona .................................. ℥ ij,

Aq. dest. ad .................................. ℥ viij.

One tablespoonful three times a day. In chronic pleurisy.—*Ib.*

**Pneumonia.**—

℞. Tinct. aconiti ..................................
Vin ipecacuhanæ .................................. aa *m* xvj,
Aq. dest .................................. ℥ ij.

One teaspoonful every hour.—*Ib.*

